# Development of Models to Predict Postoperative Complications for Hepatitis B Virus-Related Hepatocellular Carcinoma

**DOI:** 10.3389/fonc.2021.717826

**Published:** 2021-10-05

**Authors:** Mingyang Bao, Qiuyu Zhu, Tuerganaili Aji, Shuyao Wei, Talaiti Tuergan, Xiaoqin Ha, Alimu Tulahong, Xiaoyi Hu, Yueqing Hu

**Affiliations:** ^1^ State Key Laboratory of Genetic Engineering, Institute of Biostatistics, School of Life Sciences, Fudan University, Shanghai, China; ^2^ Division of Hepatobiliary and Pancreatic Surgery, Department of Surgery, The First Affiliated Hospital, Zhejiang University School of Medicine, Hangzhou, China; ^3^ Department of Surgery, Shanghai Jiaotong University School of Medicine, Shanghai, China; ^4^ Department of Hepatobiliary and Hydatid Surgery, The First Affiliated Hospital of Xinjiang Medical University, Urumqi, China; ^5^ Clinical Laboratory Diagnostics, School of Public Health, Gansu University of Chinese Medicine, Lanzhou, China; ^6^ Department of Clinical Laboratory, The 940th Hospital of Joint Logistics Support Force of Chinese People’s Liberation Army, Lanzhou, China; ^7^ Shanghai Center for Mathematical Sciences, Fudan University, Shanghai, China

**Keywords:** modeling, hepatocellular carcinoma, complications, liver resection, comprehensive complication index

## Abstract

**Background:**

Surgical treatment remains the best option for patients with hepatocellular carcinoma (HCC) caused by chronic hepatitis B virus (HBV) infection. However, there is no optimal tool based on readily accessible clinical parameters to predict postoperative complications. Herein, our study aimed to develop models that permitted risk of severe complications to be assessed before and after liver resection based on conventional variables.

**Methods:**

A total of 1,047 patients treated by hepatectomy for HCC with HBV infection at three different centers were recruited retrospectively between July 1, 2014, and July 1, 2018. All surgical complications were recorded and scored by the Comprehensive Complication Index (CCI). A CCI ≥26.2 was used as a threshold to define patients with severe complications. We built two models for the CCI, one using preoperative and one using preoperative and postoperative data. Besides, CCI and other potentially relevant factors were evaluated for their ability to predict early recurrence and metastasis. All the findings were internally validated in the Hangzhou cohort and then externally validated in the Lanzhou and Urumqi cohorts.

**Results:**

Multivariable analysis identified National Nosocomial Infections Surveillance (NNIS) index, tumor number, gamma-glutamyltransferase (GGT), total cholesterol (TC), potassium, and thrombin time as the key preoperative parameters related to perioperative complications. The nomogram based on the preoperative model [preoperative CCI After Surgery for Liver tumor (CCIASL-pre)] showed good discriminatory performance internally and externally. A more accurate model [postoperative CCI After Surgery for Liver tumor (CCIASL-post)] was established, combined with the other four postoperative predictors including leukocyte count, basophil count, erythrocyte count, and total bilirubin level. No significant association was observed between CCI and long-term complications.

**Conclusion:**

Based on the widely available clinical data, statistical models were established to predict the complications after hepatectomy in patients with HBV infection. All the findings were extensively validated and shown to be applicable nationwide. Such models could be used as guidelines for surveillance follow-up and the design of post-resection adjuvant therapy.

## Introduction

Hepatocellular carcinoma (HCC) is the most common primary liver tumor and the third leading cause of cancer-related deaths around the world. China alone accounts for more than half of the global HCC cases (1, 2). Hepatitis B virus (HBV) infection is one of the main etiologies of HCC around the world, especially in hepatitis B-prevalent regions such as sub-Saharan Africa and East Asia (3, 4). For patients suffering from chronic hepatitis B infection, the treatment of HCC is more difficult, the prognosis is worse, and the recurrence is earlier. Nevertheless, surgical treatment still remains the best option for patients with preserved liver function among a variety of treatments (5, 6).

Currently, there is no consensus regarding the optimal tool for risk stratification derived from surgically managed patients with HBV infection. Suboptimal management of perioperative period may partially lead to severe postoperative complications such as post-hepatectomy liver failure (PHLF) (7), post-hepatectomy hemorrhage (PHH), and postoperative death. For a long time, Child–Pugh scoring system, albumin–bilirubin (ALBI) grading system, and end-stage liver disease (MELD) score were the traditional basic indices of preoperative liver functions (8–10). In recent years, many novel approaches to assessing total liver function and functional remnant liver have emerged, such as indocyanine green (ICG) clearance test (10–13), liver scintigraphy, and liver stiffness measurement (LSM) by transient elastography (TE) (14–18). However, few people have access to these latest liver-specific evaluations in developing countries due to the lack of detection equipment. Hence, a prediction model that only consists of readily accessible clinical and pathological parameters is more practical to help surgeons to identify patients at risk of severe complications.

Clavien–Dindo classification was a traditional widely used grading system of surgical complications (19). On this basis, the Comprehensive Complication Index (CCI) has been proposed recently. The index integrates all recorded complications after surgery in a single formula weighted by severity and shows more sensitivity than the traditional one (20, 21).

CCI is a comprehensive measurement of short-term complications during perioperative period. In addition, tumor recurrence, a major long-term complication, deserves attention as well. Two-year duration is generally used as the cutoff to determine early or late recurrence (22, 23). Early recurrence (i.e., within 2 years after surgery) accounts for more than 70% of tumor recurrence and is considered a “real recurrence” (24). Furthermore, metastasis is also a long-term complication and an important manifestation of recurrence. Different metastatic targets represent different tumor development trends (25). However, no clear relationship has been found between short- and long-term complications after operation. In particular, prediction of tumor metastasis and correlations of metastatic sites were rarely taken into account regarding HBV-related HCC in the previous analysis.

In this study, we recruited a large number of surgically treated patients for HCC with HBV infection from different provinces in China. Comprehensive common clinical, imaging, and pathological parameters were retrospectively reviewed in order to develop and validate models of complication prediction. Two models were first developed: one included parameters available before surgery for prediction of perioperative complications measured by CCI preoperatively, and the other included all accessible variables to enhance the accuracy of prediction. Moreover, risk factors for early recurrence and metastasis were identified from all variables as well as CCI.

## Methods

### Patient Recruitment

In this national retrospective cohort study, patients were recruited from three centers in different provinces. These centers comprise Hangzhou (the First Affiliated Hospital of Zhejiang University School of Medicine, Hangzhou), Lanzhou (the 940th Hospital of Joint Logistics Support Force of Chinese People’s Liberation Army, Lanzhou), and Urumqi (the First Affiliated Hospital of Xinjiang Medical University, Urumqi).

The inclusion criteria were as follows: 1) patients undergoing liver resection for HCC diagnosed pathologically; 2) HBV surface antigen (HBsAg)- and/or HBV core antibody (HBcAb)-positive patients; 3) Child–Pugh class A or B (score ≤7) patients; 4) patients performing preoperative abdominal contrast-enhanced magnetic resonance imaging (MRI) and/or contrast-enhanced computed tomography (CT) scan; 5) anatomical and non-anatomical hepatectomies before July 1, 2018. The following exclusion criteria were also met: 1) patients undergoing more than one additional procedure in the liver; 2) patients with a history of tumors; 3) HCV-positive patients; 4) patients with incomplete clinical data; 5) patients receiving antitumor therapy before operation [i.e., transarterial chemoembolization (TACE), radiotherapy, or chemotherapy].

Through strict selection and careful data record, 1,047 eligible patients were included in total, 675 of whom were from Hangzhou (415 available survival data), 252 from Lanzhou, and 120 from Urumqi. The study obeyed the ethical guidelines of the 1975 Declaration of Helsinki and was approved by the institutional ethics committee. Informed consent was obtained from each patient before surgery.

### Data Collection

The information of patients was prospectively collected into electronic spreadsheets by each center and then retrospectively reviewed. Demographics and comorbidities of patients were obtained from detailed consultation records. Preoperative and postoperative blood was taken from the patients for laboratory tests, including marks related to HBV, a complete blood count, blood chemical analysis, and coagulation testing; all the postoperative laboratory examinations were performed 3–5 days after surgery. Serum tumor markers were also investigated, such as α-fetoprotein (AFP), carbohydrate antigen 199 (CA199), and carcinoembryonic antigen (CEA). Tumor classification and characteristics relied on imaging examination reports and pathologic results of the resected specimens. The imaging data included tumor number, diameter, capsule status, location, lymphadenectasis, esophageal varices, ascites, and cirrhosis based on preoperative contrast-enhanced MRI and/or CT. The presence of arterial enhancement and washout of lesions on each of the dynamic imaging phases were also recorded. Arterial enhancement was defined as lesions exhibiting higher signal intensity on arterial phase images (26–28). Washout was defined as lesions with higher intensity compared with the surrounding liver on any late dynamic images except the arterial phase images (26–28). The pathologic results included the microvascular invasion, tumor giant cell, status of surrounding liver tissue, and differentiation and encapsulation of tumor. In addition, pathological immunohistochemistry tests for cytokeratin 19 (CK19), cytokeratin 7 (CK7), glypican 3, and CD34 were carried out. For both imaging and pathological data, two experienced specialists independently evaluated all results respectively. Any controversies in findings between the specialists were settled by discussion and generated a unified answer. Perioperative data were derived from operative recordings. Patients were followed up in the first 2 years after surgery to observe postoperative complications and early oncological outcomes. Information collection ended on July 1, 2020.

### Measurement of Postoperative Complications and Clinical Outcomes

All surgical complications, defined as any deviation from the normal postoperative course that occurred before discharge, were recorded and scored using the CCI, ranging from 0 (uneventful course) to 100 (death). The threshold for defining patients with at least one grade III (major) complication was CCI ≥26.2 according to Clavien–Dindo classification (19, 29). This cutoff also takes into account the weight of multiple low-grade complications (e.g., grades I–II). Although these complications are not usually regarded as endpoints, they are considered to increase the postoperative experience of patients longer than a grade III complication in the CCI model. The CCI was used as the primary endpoint to assess the postoperative situation of patients.

Early recurrence of HCC was defined as the appearance of a newly detected tumor confirmed on two radiologic images within 2 years (30, 31). Relapse-free survival (RFS), the interval between liver resection and recurrence, was measured as the secondary endpoint. Among all kinds of recurrence, new tumor not located as the same as the primary one was diagnosed as metastasis. The metastasis was further divided into six categories depending on the location where tumor was newly discovered (lung, liver, abdomen, bone, lymph nodes, and brain).

### Statistical Analyses

Continuous variables were described as medians with interquartile range (IQR), and nonparametric Mann–Whitney U tests were applied for statistical significance between high CCI (≥26.2) group and low CCI (<26.2) group. For categorical variables, we expressed the numbers and percentages of patients in each category. Proportions were compared using the χ^2^ test, with Yates’ correction or Fisher’s exact test. Serum HBV DNA, AFP, CA199, CA125, and ferritin were natural log transformed due to high skewness to the right.

The preoperative and postoperative CCI After Surgery for Liver tumor (CCIASL) models were built to predict the risk of developing high CCI on the Hangzhou dataset and then internally validated through bootstrap resampling. Subsequently, external validation of the two models was conducted on datasets from Lanzhou and Urumqi. One of the models, CCIASL-pre model, was based on preoperative predictors available before surgery; the other model, CCIASL-post model, was constructed on all available parameters.

Univariable logistic regression analyses were performed to screen potentially relevant variables. Those that reached p < 0.05 at univariable analysis were included in the multivariable logistic regression, and two-way stepwise strategy was applied for predictor selection. A number of potentially clinically plausible interactions were also taken into account during this process. The candidate multivariable regression model was built when Akaike information criterion (AIC) reached the minimum. We retained the statistically significant predictors in the final model. The risk score for prediction of high CCI was defined as the weight sum of the value of those significant parameters, and the weights were model β-estimates. The samples from each cohort were cut to high-risk and low-risk groups based on the median of risk score. Boxplots were drawn to compare risk differences between the two groups. The discrimination performance of the two models in the derivation and validation sets was then measured by the Harrell’s concordance index (C-index) and the receiver operating characteristic (ROC) curves. A relatively corrected C-index was also calculated by 1,000 bootstrap resampling for internal validation. Moreover, we made comparisons between two models. Net reclassification improvement (NRI) and integrated discrimination improvement (IDI) were computed using the category-free approach with 1,000 bootstrap replications to estimate the 95% confidence interval (CI) (32). The significant factors related to high CCI in the two models were used to construct nomograms. Calibration curves were subsequently drawn to assess the Hosmer–Lemeshow goodness of fit (33). Bootstrapping was used for each model to get bias-corrected (overfitting-corrected) estimates of predicted vs. observed values. As a reference line, the diagonal represents the best prediction. We also performed a decision curve analysis to determine whether our established nomogram was suitable for clinical utility by estimating the net benefits at different threshold probabilities.

Moreover, univariable Cox regression was applied to identify the association between CCI and early recurrence of HCC. The samples were stratified into various subgroups based on a number of important HCC-related markers, surgical factors, and tumor characteristics. Subgroup analysis was then conducted to explore possible correlations in each specific group of HCC patients. To identify more predictive factors for RFS, significant variables at univariable analysis were included to build a multivariable Cox regression model by both-direction stepwise selection. The proportional hazards assumption of the models was tested by examining the plots of scaled Schoenfeld residuals against time for each variable in the models. The patients were grouped into high risk and low risk according to median of the risk score, which was computed similar to the logistic regression described above. Kaplan–Meier (KM) survival analysis was performed to estimate the prediction value of the model. Survival nomogram was then constructed based on the significant factors in the multivariable Cox regression model. The predictive accuracy of the models was quantified through the concordance index (C-index) and time-dependent ROC curves with area under the curve (AUC). The level of agreement between the predicted probabilities and the actual possibility of early recurrence was measured by calibration plots. Model discrimination was performed both in the derivation cohort and in the validation cohorts.

To further explore the association between CCI and metastasis of HCC, univariable generalized linear model (GLM, here was logistic regression) was used. To identify more factors related to metastasis, significant variables at univariable analysis were included to build a multivariable GLM by both forward and backward stepwise selection. Given that tumor metastasis involved multiple locations, generalized estimation equation (GEE) was established to obtain more robust coefficients of the variables chosen by GLM selection and explore the correlations between different metastasis sites. The unstructured working correlation matrix was adopted due to the uncertainty of associations between various metastasis sites. The external validation set in this part was composed of Lanzhou and Urumqi cohorts.

All statistical tests were two-tailed, and differences were considered significant at a p-value <0.05. All statistical analyses were performed using R 3.6.1 software (R Foundation for Statistical Computing, Vienna, Austria) and SPSS 24.0 (SPSS, Chicago, USA).

## Results

### Patient Characteristics

Baseline features and postoperative complications were collected from 675 patients who were infected with HBV and received liver section for HCC in the Hangzhou cohort (**Table 1**). The median age was 56 years (IQR: 48–63), and the majority of patients were male (81.19%). Nearly all of the patients were Child–Pugh A (98.8%) and had positive HBsAg and HBcAb (99.4%). A total of 92 patients was considered high CCI (≥26.2), accounting for 13.6% of the whole population. Perioperative complications of patients with high CCI in the Hangzhou cohort were shown in detail in **Table 2**. This proportion was 13.3% in the Urumqi cohort and 7.9% in the Lanzhou cohort. Compared with patients with low CCI, those with high CCI were more likely from the worse physical condition and higher surgery risk according to the National Nosocomial Infections Surveillance (NNIS) index (p < 0.001), American Society of Anesthesiologists (ASA) score (p = 0.009), and Barcelona Clinic Liver Cancer (BCLC) stage (p < 0.001). Patients with high CCI had a longer hospital stay and a larger amount of ascites than those with low CCI. The median HBV-DNA level of all patients was 11.13 IU/ml (after natural log transformation), and insignificantly level increase was observed in high CCI group. High CCI patients also showed numerous differences in preoperative laboratory results, such as proportion of various blood cells, levels of bilirubin and enzymes, and coagulation time. Moreover, these differences became more significant after surgery. Based on imaging examination and pathological analysis, we discovered that tumor number, maximum tumor size, and conditions of hepatic capsule and surrounding satellite nodules were significantly different between the two groups. Of 415 patients with survival data, nearly half relapsed within 2 years and 65 metastasized. The average RFS of all was 16.29 months. No significant differences in clinical outcomes between the two groups of patients were observed. More detailed information of patients was shown in **Table S1**.

**Table 1:** Characteristics of study population and grouped by comprehensive complication index (CCI).

Variables	All patients (n=675)	CCI≥26.2 (n=92)	CCI<26.2 (n=583)	P
**Baseline features**				
Age, years, median [IQR]	56.00 [48.00, 63.00]	57.50 [48.00, 64.25]	56.00 [48.00, 63.00]	0.326
Male, n (%)	548 (81.19)	469 (80.45)	79 (85.87)	0.274
NNIS index, n (%)				<0.001
0	196 (29.0)	11 (12.0)	185 (31.7)	
1	462 (68.4)	75 (81.5)	387 (66.4)	
2	17 (2.5)	6 (6.5)	11 (1.9)	
Antivirus treatment, n (%)	168 (24.9)	23 (25.0)	145 (24.9)	>0.999
Child Pugh, n (%)				0.299
A	667 (98.8)	90 (97.8)	577 (99.0)	
B	8 (1.2)	2 (2.2)	6 (1.0)	
BCLC stage, n (%)				<0.001
0	424 (62.8)	46 (50.0)	378 (64.8)	
A1	153 (22.7)	20 (21.7)	133 (22.8)	
A2	12 (1.8)	2 (2.2)	10 (1.7)	
A3	65 (9.6)	15 (16.3)	50 (8.6)	
A4	21 (3.1)	9 (9.8)	12 (2.1)	
**Hepatitis B marks**				
Hepatitis B Virus DNA, IU/mL, median [IQR]	11.13 [0.00, 16.71]	12.33 [0.00, 17.99]	11.03 [0.00, 16.45]	0.187
Hepatitis B core antibody, S/CO, median [IQR]	10.62 [9.41, 11.73]	10.70 [9.40, 11.94]	10.60 [9.42, 11.70]	0.626
Hepatitis B surface antigen positive, n (%)	671 (99.4)	579 (99.3)	92 (100.0)	>0.999
Hepatitis B e antigen positive, n (%)	154 (22.8)	129 (22.1)	25 (27.2)	0.287
Hepatitis B e antibody positive, n (%)	191 (28.3)	167 (28.6)	24 (26.1)	0.709
Hepatitis B surface antibody positive, n (%)	190 (28.1)	160 (27.4)	30 (32.6)	0.319
**Radiology findings**				
Multiple lesions, n (%)	86 (12.8)	24 (26.1)	62 (10.7)	<0.001
Maximum tumor size, cm, median [IQR]	5.00 [3.50, 7.50]	6.00 [4.38, 8.00]	5.00 [3.50, 7.50]	0.041
Hepatic capsule, n (%)				0.042
Normal	526 (78.2)	66 (71.7)	460 (79.2)	
Invaded	89 (13.2)	20 (21.7)	69 (11.9)	
Attached	58 (8.6)	6 (6.5)	52 (9.0)	
Cirrhosis, n (%)	389 (57.9)	51 (55.4)	338 (58.3)	0.650
Ascites, n (%)				0.110
No	555 (83.0)	74 (81.3)	481 (83.2)	
Mild	107 (16.0)	14 (15.4)	93 (16.1)	
Moderate	6 (0.9)	3 (3.3)	3 (0.5)	
Surrounding satellite nodules, n (%)	98 (14.6)	22 (23.9)	76 (13.1)	0.010
**Preoperative laboratory findings (median [IQR])**				
Leukocyte, 10^9/L	6.90 [5.30, 8.70]	7.35 [5.12, 9.54]	6.78 [5.22, 8.77]	0.097
Basophil, %	0.30 [0.20, 0.50]	0.30 [0.20, 0.50]	0.30 [0.20, 0.50]	0.331
Erythrocyte, 10^12/L	4.64 [4.27, 5.00]	4.46 [4.20, 4.85]	4.66 [4.30, 5.01]	0.015
Albumin-globulin ratio	1.60 [1.30, 1.80]	1.50 [1.30, 1.80]	1.60 [1.40, 1.80]	0.478
Alanine aminotransferase, U/L	29.00 [20.00, 45.00]	29.00 [19.00, 46.00]	29.00 [21.00, 45.00]	0.862
Aspartate aminotransferase, U/L	32.00 [25.00, 44.00]	30.50 [25.00, 47.00]	32.50 [25.00, 44.00]	0.730
Total bilirubin, μmol/L	13.00 [10.00, 17.00]	14.00 [10.50, 17.50]	13.00 [10.00, 17.00]	0.168
Gamma-glutamyltransferase, U/L	59.00 [34.00, 109.00]	82.50 [40.50, 155.25]	57.00 [34.00, 105.00]	0.004
Total cholesterol, mmol/L	3.91 [3.45, 4.57]	3.71 [3.11, 4.50]	3.96 [3.51, 4.58]	0.010
Potassium, mmol/L	4.16 [3.92, 4.40]	4.04 [3.88, 4.31]	4.18 [3.94, 4.41]	0.005
Thrombin time, s	18.60 [17.60, 19.70]	18.30 [17.30, 19.20]	18.70 [17.60, 19.70]	0.031
**Surgical features**				
Laparoscopy surgery, n (%)	98 (14.6)	6 (6.5)	92 (15.8)	0.017
Open surgery, n (%)	577 (85.7)	87 (94.6)	490 (84.3)	0.006
Minor hepatectomy, n (%)	522 (77.6)	66 (71.7)	456 (78.5)	0.178
Major hepatectomy, n (%)	149 (22.1)	26 (28.3)	123 (21.2)	0.138
Pringle Maneuver, n (%)	76 (11.3)	11 (12.0)	65 (11.2)	0.859
Intraoperative blood transfusion, n (%)	44 (6.6)	10 (11.0)	34 (5.9)	0.107
Intraoperative blood loss, mL, median [IQR]	200.00 [100.00, 400.00]	300.00 [200.00, 500.00]	200.00 [100.00, 400.00]	<0.001
**Pathologic characteristics**				
Tumor differentiation, n (%)				0.734
Well	6 (0.9)	0 (0.0)	6 (1.0)	
Moderate	32 (4.8)	6 (6.5)	26 (4.5)	
Moderate to Poor	568 (84.9)	78 (84.8)	490 (84.9)	
Poor	63 (9.4)	8 (8.7)	55 (9.5)	
Satellites, n (%)	62 (9.2)	8 (8.7)	54 (9.3)	>0.999
Microvascular invasion, n (%)	215 (31.9)	31 (33.7)	184 (31.7)	0.719
CD34, n (%)	479 (97.8)	66 (100.0)	413 (97.4)	0.374
Cytokeratin 19, n (%)	198 (33.6)	27 (35.5)	171 (33.3)	0.698
**Postoperative laboratory findings (median [IQR])**				
Leukocyte, 10^9/L	7.10 [5.50, 9.20]	7.75 [5.47, 10.45]	7.00 [5.50, 9.00]	0.033
Basophil, %	0.30 [0.20, 0.40]	0.30 [0.20, 0.50]	0.20 [0.20, 0.40]	0.022
Erythrocyte, 10^12/L	3.65 [3.23, 4.05]	3.44 [3.02, 3.82]	3.68 [3.30, 4.07]	0.001
Alanine aminotransferase, U/L	65.00 [45.00, 96.50]	59.00 [42.50, 98.50]	67.00 [45.00, 96.25]	0.337
Aspartate aminotransferase, U/L	34.00 [26.00, 48.00]	36.00 [26.00, 50.00]	33.50 [26.00, 47.00]	0.723
Total bilirubin, μmol/L	19.00 [14.00, 26.00]	22.60 [15.00, 36.00]	19.00 [14.00, 25.00]	<0.001
Gamma-glutamyltransferase, U/L	72.00 [43.00, 116.00]	89.00 [46.50, 142.75]	71.00 [43.00, 114.00]	0.031
Total cholesterol, mmol/L	2.61 [2.11, 3.15]	2.42 [1.90, 2.96]	2.62 [2.15, 3.16]	0.034
**Perioperative situation (median [IQR])**				
POD 1 ascites volume, mL	150.00 [80.00, 250.00]	200.00 [100.00, 300.00]	130.00 [80.00, 220.00]	0.001
Hospital stay, days	19.00 [16.00, 24.00]	27.00 [22.00, 32.00]	19.00 [15.00, 23.00]	<0.001
Postoperative hospital stay, days	10.00 [8.00, 13.00]	16.00 [13.00, 20.00]	10.00 [8.00, 12.00]	<0.001
Clinical outcomes **				
Recurrence within 2 years, n (%)	199 (47.95)	24 (43.64)	175 (48.61)	0.587
Recurrence-free survival, months (mean±SD)	16.29±8.93	17.15±8.94	16.16±8.93	0.447
Metastasis, n (%)	65 (15.66)	7 (12.73)	58 (16.11)	0.657

NNIS, National Nosocomial Infection Surveillance; ASA, American Society of Anesthesiologists; BCLC, Barcelona Clinic Liver Cancer; POD, postoperative day; IQR, interquartile range; SD, standard deviation. * data was natural log transformed; ** the total number of patients available to survival data was 415.

**Table 2:** Postoperative complications of patients with comprehensive complication index (CCI) ≥26.2 and their grade of severity.

Grading of complications (CCI≥26.2)	No. of complications	Details of complications
Grade I	64	abdominal infection=9; electrolytes=2; fever=1; pneumonia=45; wound infection=7
Grade II	57	abdominal abscess=1; ascites=7; atrial fibrillation=3; biliary leak=7; blood oozing=5; blood transfusion=24; delirium=1; gastroplegia=1; hemoperitoneum=1; ileus=2; pleural effusion=1; thrombosis=3
Grade IIIa	79	abdominal abscess=1; ascites=13; bleeding=1; bleeding/hematoma=1; perihepatic ascites=8; pleural effusion=51; thrombosis=2; wound infection=2
Grade IIIb	4	hemoperitoneum=3; bleeding/hematoma=1
Grade IVa	9	acute liver failure=3; pulmonary embolism=3; respiratory insufficiency=3
Grade IVb	1	shock=1
Grade V	2	MOF=2

MOF, multi-organ failure.

### Construction of the Model Predicting Severe Perioperative Complications

The result of univariable logistic regression analysis revealed that NNIS index, ASA score (III *vs*. I), BCLC stage (A4 *vs*. 0 and A4 *vs*. 0), tumor number, hepatic capsule (invaded *vs*. normal), surrounding satellite nodules, surgical approach, postoperative day (POD) 1 ascites volume, and intraoperative blood loss were significantly relevant with high CCI (**Table 3**). From the results of laboratory tests, erythrocyte count, total protein (TP), alkaline phosphatase (ALP), gamma-glutamyltransferase (GGT), and total cholesterol (TC) were discovered to be significantly associated with high CCI both before and after operation. Notably, the correlation between proportion of basophils in leukocytes and high CCI became significant in those experiencing surgery. The change of significance was observed in the relation of total bilirubin and high CCI as well.

**Figure 1:**
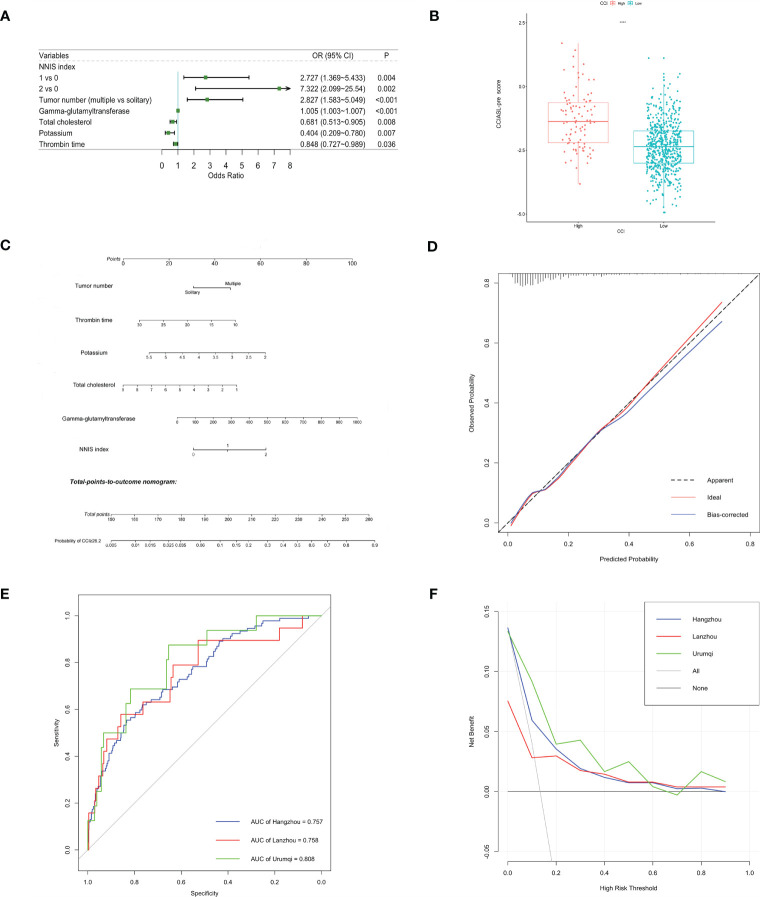
Construction and validation of the CCIASL-pre model. **(A)** Forest plot of predictors for CCI≥26.2 based on the result of multivariate analysis. **(B)** Boxplot of the CCIASL-pre risk score between high CCI group and low CCI group. Significant difference was observed. ****: P<0.0001. **(C)** Nomogram to predict probability of CCI≥26.2 in HCC patients. **(D)** Calibration curve for the nomogram to predict probability of CCI≥26.2 in the derivation cohort. The x-axis represents the predicted CCI≥26.2 probability and the y-axis denotes the actual proportion of CCI≥26.2. The black diagonal line indicates the best prediction. The red ideal line represents the uncorrected performance of the nomogram while the blue line shows the bias-corrected performance. **(E)** Receiver operating characteristic (ROC) curves in the derivation and validation cohorts. Corresponding area under curves (AUC) in the Hangzhou, Lanzhou and Urumqi were 0.757, 0.758 and 0.808, respectively. **(F)** Decision curve for the predictive nomogram. The net benefits were measured at different threshold probabilities. The blue, red and green lines represent the predictive ability of nomogram in the Hangzhou, Lanzhou and Urumqi cohorts, respectively. The gray line represents the assumption that all patients have severe complications. The black line represents the assumption that no patients have severe complications.

**Figure 2:**
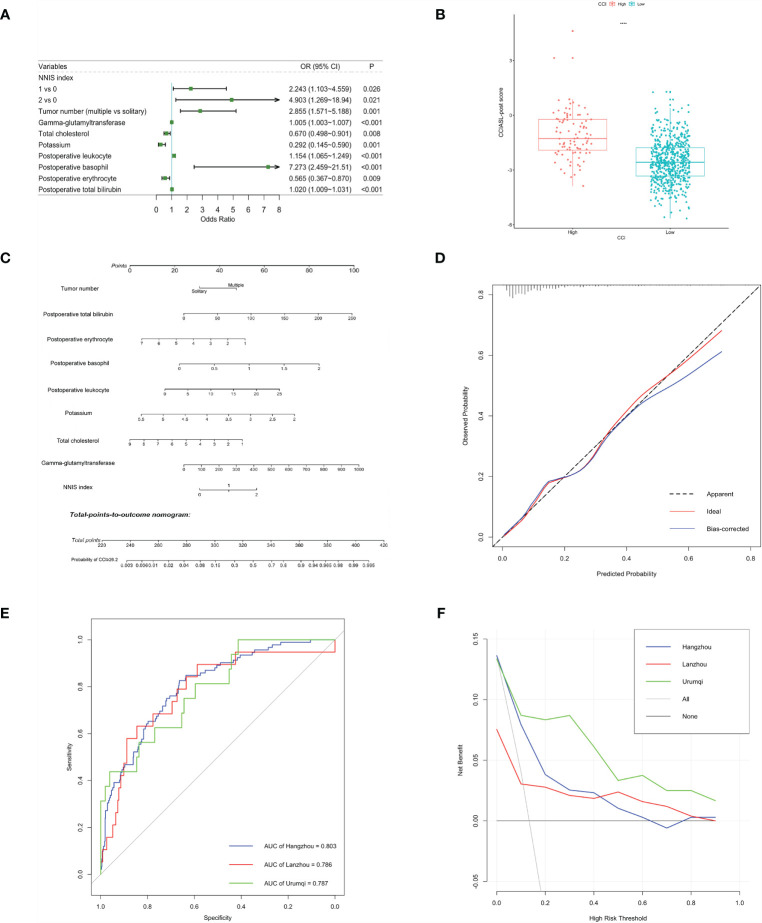
Construction and validation of the CCIASL-post model. **(A)** Forest plot of predictors for CCI≥26.2 based on the result of multivariate analysis. **(B)** Boxplot of the CCIASL-post risk score between high CCI group and low CCI group. Significant difference was observed. ****: P<0.0001. **(C)** Nomogram to predict probability of CCI≥26.2 in HCC patients. **(D)** Calibration curve for the nomogram to predict probability of CCI≥26.2 in the derivation cohort. The x-axis represents the predicted CCI≥26.2 probability and the y-axis denotes the actual proportion of CCI≥26.2. The black diagonal line indicates the best prediction. The red ideal line represents the uncorrected performance of the nomogram while the blue line shows the bias-corrected performance. **(E)** Receiver operating characteristic (ROC) curves for severe complications in the derivation and validation cohorts. Corresponding area under curves (AUC) in the Hangzhou, Lanzhou and Urumqi were 0.803, 0.786 and 0.787, respectively. **(F)** Decision curve for the predictive nomogram. The net benefits were measured at different threshold probabilities. The blue, red and green lines represent the predictive ability of nomogram in the Hangzhou, Lanzhou and Urumqi cohorts, respectively. The gray line represents the assumption that all patients have severe complications. The black line represents the assumption that no patients have severe complications.

After a multistep selection of significant variables at univariable analysis available before surgery, six of them were employed for building CCIASL-pre model, including NNIS index, tumor number, GGT, TC, potassium, and thrombin time (TT) (**Figure 1A**). The risk score formula was shown at the bottom of **Table 3**. As shown in the boxplot, patients with high CCI were scored significantly higher (**Figure 1B**). Based on the independent predictors in the final multivariable model, a nomogram was constructed to visualize the relationship with probability of high CCI (**Figure 1C**). In addition, another multivariable logistic regression model was developed based on all variables significant at univariable analysis to construct CCIASL-post. Five parameters of the CCIASL-pre model, namely, NNIS index, tumor number, GGT, TC, and potassium, maintained independence. On this basis, another four postoperative laboratory findings were identified, including leukocyte count, basophil, erythrocyte count, and total bilirubin (**Figure 2A**). Similar to the CCIASL-pre model, postoperative model formula of risk score was shown at the bottom of **Table 3**, and boxplot was drawn to display risk differences between two groups classified by the median risk score (**Figure 2B**). Nine independent predictors of CCIASL-post model were integrated in the nomogram (**Figure 2C**).

**Table 3:** Uni- and multivariate logistics regression analysis of predictors for high Comprehensive Complication Index (CCI≥26.2).

	Univariate analysis			Multivariate analysis					
				*Without postoperative results*			*With postoperative results*		
Variables	OR	95% CI	P	OR	95% CI	P	OR	95% CI	P
**Baseline factors**									
Sex (male vs female)	1.477	0.794-2.749	0.218						
Age (years)	1.008	0.988-1.029	0.448						
NNIS index									
1 vs 0	3.259	1.690-6.285	<0.001	2.727	1.369-5.433	0.004	2.243	1.103-4.559	0.026
2 vs 0	9.174	2.859-29.44	<0.001	7.322	2.099-25.54	0.002	4.903	1.269-18.94	0.021
ASA score									
II vs I	1.312	0.759-2.268	0.331						
III vs I	3.576	1.601-7.984	0.002						
Child Pugh (B vs A)	2.137	0.425-10.75	0.357						
BCLC stage									
A1 vs 0	1.236	0.705-2.165	0.460						
A2 vs 0	1.643	0.349-7.734	0.530						
A3 vs 0	2.465	1.283-4.737	0.007						
A4 vs 0	6.163	2.464-15.42	<0.001						
**Tumor characteristics**									
Tumor number (multiple vs solitary)	2.943	1.724-5.024	<0.001	2.827	1.583-5.049	<0.001	2.855	1.571-5.188	0.001
Hepatic capsule									
Invaded vs Normal	2.020	1.153-3.539	0.014						
Attached vs Normal	0.804	0.332-1.946	0.629						
Surrounding satellite nodules (present vs absent)	2.088	1.221-3.570	0.007						
**Perioperative situation**									
Laparoscopy surgery (yes vs no)	0.371	0.157-0.874	0.023						
Open surgery (yes vs no)	3.231	1.277-8.179	0.013						
POD 1 ascites volume (mL)	1.002	1.001-1.003	0.001						
Intraoperative blood loss (mL)	1.001	1.000-1.001	0.003						
**Preoperative laboratory results**									
Leukocyte (10^9/L)	1.068	0.936-1.136	0.074						
Basophil (%)	1.443	0.678-3.071	0.342						
Erythrocyte (10^12/L)	0.623	0.419-0.929	0.020						
Total protein (g/L)	0.959	0.926-0.992	0.016						
Alkaline phosphatase (U/L)	1.007	1.003-1.012	0.001						
Total bilirubin (μmol/L)	1.016	0.981-1.052	0.379						
Gamma-glutamyltransferase (U/L)	1.004	1.002-1.006	<0.001	1.005	1.003-1.007	<0.001	1.005	1.003-1.007	<0.001
Total cholesterol (mmol/L)	0.735	0.566-0.954	0.021	0.681	0.513-0.905	0.008	0.670	0.498-0.901	0.008
Potassium (mmol/L)	0.465	0.257-0.839	0.011	0.404	0.209-0.780	0.007	0.292	0.145-0.590	0.001
Thrombin time (s)	0.853	0.738-0.986	0.032	0.848	0.727-0.989	0.036			
Carbohydrate antigen199 (kU/L)	1.160	1.018-1.323	0.026						
**Postoperative laboratory results**									
Leukocyte (10^9/L)	1.098	1.026-1.175	0.007				1.154	1.065-1.249	<0.001
Basophil (%)	4.553	1.742-11.90	0.002				7.273	2.459-21.51	<0.001
Erythrocyte (10^12/L)	0.570	0.391-0.832	0.004				0.565	0.367-0.870	0.009
Total protein (g/L)	0.946	0.910-0.983	0.005						
Alkaline phosphatase (U/L)	1.007	1.003-1.012	0.002						
Total bilirubin (μmol/L)	1.020	1.010-1.029	<0.001				1.020	1.009-1.031	<0.001
Gamma-glutamyltransferase (U/L)	1.003	1.001-1.005	0.003						
Total cholesterol (mmol/L)	0.687	0.495-0.954	0.025						
CCIASL-pre score = 1.013 × NNIS index (=1) or 1.002 × NNIS index (=2) + 0.005 × Gamma-glutamyltransferase (U/L) – 0.382 × Total cholesterol (mmol/L) – 0.920 × Potassium (mmol/L) – 0.172 × Thrombin time (s) + 1.037 × Tumor number (0: solitary; 1: multiple) + 4.775									
CCIASL-post score = 0.847 × NNIS (=1) or 0.824 × NNIS (=2) + 0.005 × Gamma-glutamyltransferase (U/L) – 0.407 ×Total cholesterol (mmol/L) + 0.132 × Leukocyte (10^9/L)* + 2.015 × Basophil (%)* – 0.497 ×Erythrocyte (10^12/L)* + 0.0019 × Total bilirubin (μmol/L)* + 1.063 × Tumor number (0: solitary; 1: multiple) +3.150									

NNIS, National Nosocomial Infection Surveillance; ASA, American Society of Anesthesiologists; BCLC, Barcelona Clinic Liver Cancer; POD, postoperative day; OR, odds ratio; CI, confidence interval; CCIASL, CCI After Surgery for Liver tumor. *postoperative data.

### Internal and External Assessment of the CCIASL Model

Both CCIASL models were first assessed internally in the Hangzhou cohort. As to CCIASL-pre model, the Hosmer–Lemeshow test did not indicate evidence of poor fit (p = 0.769), and the calibration plot also showed a good prediction capability (**Figure 1D**). The Harrell’s C-index, equivalent to AUC on ROC curves, was 0.757 (95% CI: 0.704–0.810) and corrected to be 0.767 (95% CI: 0.715–0.817) through bootstrapping validation, which indicated good discriminatory performance of the model (**Figure 1E** and **Table 4**). Additionally, the decision curve showed that making use of this model for predicting the probability of high CCI would gain more net benefits than an all-or-none patient intervention scheme if the threshold probability was less than 88%, which suggested a high potential for clinical application (**Figure 1F**). In the external validation cohorts, good discrimination and prediction ability were also observed, although calibration plots showed a slight deterioration, which was most pronounced in the Urumqi cohort (**Figure S1**). In terms of clinical usefulness, the Lanzhou cohort displayed better if threshold probability was more than 60%, while the Urumqi cohort performed better if threshold probability was less than 60%.

**Table 4:** Performance measurement and comparison of the CCIASL models.

Measure of discrimination	Cohort	CCIASL-pre	95% CI	CCIASL-post	95% CI	P
Harrell’s c-index	Hangzhou	0.757	0.704-0.810	0.803	0.756-0.850	
	Hangzhou (bootstrap) *	0.767	0.715-0.817	0.811	0.764-0.859	
	Lanzhou	0.758	0.632-0.884	0.786	0.671-0.901	
	Urumqi	0.808	0.698-0.919	0.787	0.670-0.905	
Net reclassification improvement	Hangzhou	Reference		-0.006	-0.061-0.048	0.820
	Lanzhou	Reference		-0.011	-0.107-0.084	0.814
	Urumqi	Reference		0.154	-0.086-0.393	0.208
Integrated discrimination improvement	Hangzhou	Reference		0.005	-0.019-0.028	0.688
	Lanzhou	Reference		-0.006	-0.018-0.006	0.342
	Urumqi	Reference		0.024	-0.103-0.151	0.712

CCIASL, Comprehensive Complication Index After Surgery for Liver tumor. *The Harrell’s c-index was internally validated by 1000 bootstrap resampling.

A better result was obtained regarding discriminatory performance of CCIASL-post model (**Figures 2D, E**). The C-indices reached around 0.8 in both the derivation cohort and validation cohorts. As shown in **Figure 2F**, the CCIASL-post model performed better in the validation cohorts, suggesting that its clinical practice was wider than that of the CCIASL-pre model, although the postoperative model would only gain more benefits if the threshold probability was less than 70% in the Hangzhou cohort. However, the results of reclassification analyses detected no significant differences between performance of the two models in all cohorts.

### Prediction for Early Recurrence and Metastasis

**Figure 3:**
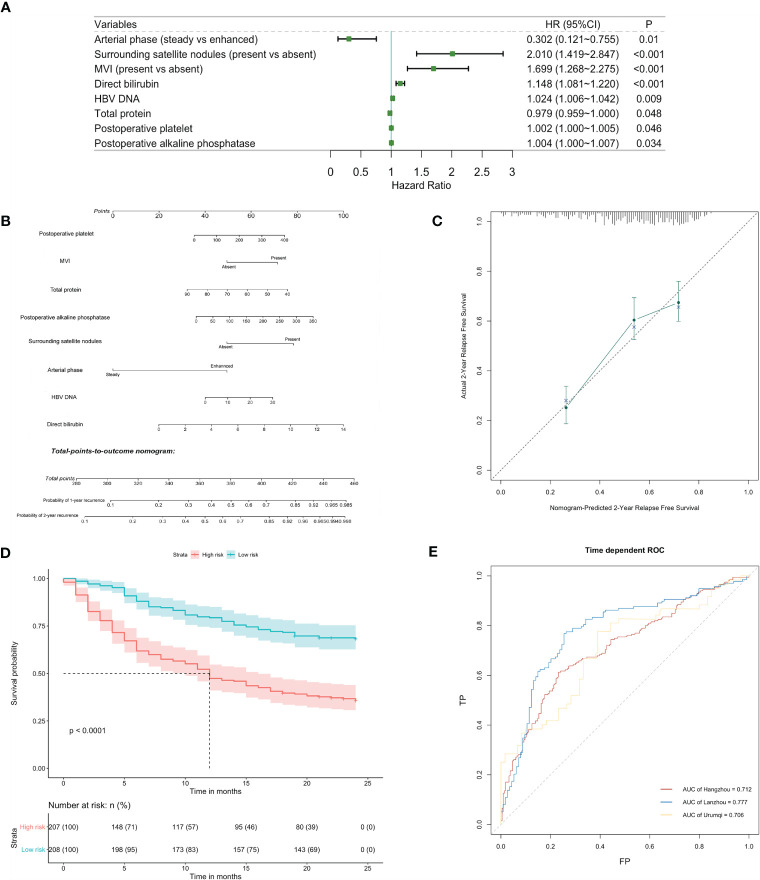
Identification and validation of risk factors for early recurrence. **(A)** Forest plot of predictors for early recurrence based on the result of multivariate analysis. **(B)** Nomogram to predict probability of early recurrence. **(C)** Calibration curve for the nomogram to predict probability of early recurrence in the derivation cohort. **(D)** Kaplan-Meier survival curve (high-risk vs low-risk patients) for relapse-free survival in the derivation cohort. **(E)** Time-dependent receiver operating characteristic (ROC) curves for relapse-free survival at 2 years in the derivation and validation cohorts. Corresponding area under curves (AUC) in the Hangzhou, Lanzhou and Urumqi were 0.712, 0.777 and 0.706, respectively.

Among all variables analyzed, CCI (high *vs*. low) was found not to be potentially relevant with early recurrence in the univariable analysis (hazard ratio = 0.862, 95% CI: 0.563–1.321, p = 0.496) (**Table S2**). The result was consistent in subgroup analysis, which demonstrated that perioperative complications after liver resection in patients with HBV infection had little association with the prediction for early relapse (**Table S3**). Nevertheless, by stepwise analysis, independent parameters were identified to be correlated with early recurrence, including HBV DNA, arterial phase, surrounding satellite nodules, microvascular invasion, preoperative TP, preoperative direct bilirubin, postoperative platelets, and postoperative ALP (**Figure 3A**). We did not detect any significant violation of the proportional hazard assumption, assessed by scaled Schoenfeld residuals on functions of time. The prognostic nomogram for early recurrence was shown in **Figure 3B**, which had a good prediction capability with a C-index of 0.701 (95% CI: 0.672–0.740) and the bootstrap estimate of C-index was 0.710 (95% CI: 0.649–0.776). The estimator in the validation cohort reached around 0.7, indicating that the prediction performance of the model was stable. The calibration plot showed an overall good agreement between the nomogram-predicted RFS and observed outcome (**Figure 3C**). This was also the case for the Lanzhou validation sets, but the deviation was larger in the Urumqi cohort (**Figures S2A, B**). Furthermore, time-dependent ROC analysis indicated that the prediction accuracies exceeded 0.7 in all cohorts (**Figure 3E**). KM survival curves displayed significantly poorer RFS of high-risk group categorized by the median risk score in the derivation and validation sets (p < 0.0001; **Figure 3D**; **Figures S2C, D**).

To analyze impact factors on early relapse in more detail, we identified 65 patients with tumor metastasis from those experiencing recurrence in the internal cohort. The locations of metastasis in more than two cases were lung, liver, abdomen, bone, lymph nodes, and brain. Lung was the most common metastatic site, accounting for 7.23% of the entire population (**Table S4**). In the univariable analysis, CCI showed no relationship with metastasis (odds ratio = 0.759, 95% CI: 0.327–1.761, p = 0.521), which was consistent with previous findings. However, a total of 20 features were identified to be found potentially associated with metastasis (**Table S5**). These variables were then included in the multivariable model to select independent factors. As a result, fasting blood glucose (FBG) and TT decreased the odds of metastasis, while postoperative TP, intraoperative blood loss, maximum tumor size, surrounding satellite nodules, and tumor encapsulation increased the odds. In the external set, GEE analysis revealed that TT was a protective factor for metastasis, and absence of tumor encapsulation increased the risk of metastasis (**Table 5**). The correlations between the locations of tumor metastasis were uncertain according to working correlation matrix (**Table S6**), but whether negative or positive, the correlation was weak.

**Table 5:** Selection of variables associated with tumor metastasis.

Variables	Multivariate GLM Selection				GEE Internal Validation				GEE External Validation			
	Coefficient	Standard Error	OR (95% CI)	P	Coefficient	Standard Error	OR (95% CI)	P	Coefficient	Standard Error	OR (95% CI)	P
Fasting blood glucose (mmol/L)	-0.387	0.166	0.679 (0.490-0.940)	0.020	-0.363	0.151	0.696 (0.518-0.934)	0.016				
Thrombin time (s)	-0.262	0.11	0.769 (0.620-0.954)	0.017	-0.261	0.109	0.771 (0.623-0.953)	0.016	-0.113	0.032	0.893 (0.839-0.951)	<0.001
Postoperative total protein (g/L)	0.079	0.025	1.082 (1.031-1.136)	0.001	0.076	0.038	1.079 (1.002-1.163)	0.045				
Intraoperative blood loss (mL)	0.001	<0.001	1.001 (1.000-1.002)	0.003								
Maximum tumor size (cm)	0.122	0.043	1.130 (1.038-1.229)	0.005								
Surrounding satellite nodules (yes vs no)	0.938	0.345	2.555 (1.299-5.026)	0.007	0.674	0.314	1.962 (1.060-3.630)	0.032				
Tumor encapsulation (absent vs complete)	1.156	0.442	3.178 (1.336-7.557)	0.009	1.414	0.388	4.111 (1.921-8.795)	<0.001	0.359	0.174	1.432 (1.019-2.013)	0.039

GLM, Generalized linear model; GEE, Generalized estimation equation; OR, Odds ratio; CI, Confidence interval.

## Discussion

Two models (CCIASL-pre and CCIASL-post) that enable risk assessment of perioperative complications before and after surgery have been derived and validated in a large national multicenter study of patients who underwent liver resection for HCC. Considering China is a hepatitis B-prevalent region, the models were constructed based on patients infected with HBV. Correspondingly, two predictive nomograms made assessments of complications more accurate by quantitatively establishing the relationship between values of predictors and probability of high CCI in a personalized way. This is a user-friendly tool for surgeons in clinical decision-making. The models are also suitable for simple application by stratifying surgical candidates into different risk levels based on risk scores.

Attentive postoperative management forms a core element in speeding recovery and reducing the chance of complications after hepatectomy, especially in cirrhotic livers with HBV infection. Postoperative bile leakage, ascites, hemorrhage, liver failure, and intra-abdominal abscesses were major short-term complications after liver resection, and the incidence of hepatectomy-related PHLF ranged from 0% to 43.1% that was frequently associated with mortality (34). In recent studies, LSM by emerging imaging examination, especially transient elastography and magnetic resonance elastography (35, 36), has been demonstrated to quantify the status of fibrosis and reflect the liver function in patients with HCC in order to promote perioperative management. Serenari et al. (18) reported a preoperative prediction of high CCI in patients with resectable HCC mainly based on LSM values. However, the accuracy of this transient elastography (FibroScan^®^) has been challenged by the risk of overestimating LSM. Actually, LSM is influenced by several other confounding factors such as age, obesity or high body mass index (BMI) and serum alanine aminotransferase (ALT), and total bilirubin levels (18, 37). Furthermore, recent studies showed that both acute hepatitis B (38) and chronic HBV infection (39) could increase the LSM value without significant fibrosis, indicating its less diagnostic accuracy in patients with HBV infection. Moreover, these measurements have not been easily accessible in developed countries, suggesting restricted practice in the clinics. Notably, our models are the first to be built solely on simple and readily available clinicopathological parameters. NNIS index, GGT, TC, potassium, and tumor number were identified to be significantly associated with severe complications in both models, indicating that these five preoperative variables possessed strong predictive capabilities for severe complications.

The NNIS system was first proposed by the Centers for Disease Control and Prevention (CDC), Atlanta, in the 1970s to predict surgical site infections (SSIs), which were considered to be the third most frequent nosocomial infection, occurring in 14%–16% of hospital inpatients (40, 41). A total of three risk factors have been evaluated in the NNIS system containing the status of surgical wound, the anesthesia score, and the procedure duration. As a nosocomial infection surveillance system, NNIS index is also an essential component in the Study of the Efficacy of Nosocomial Infection Control (SENIC) index (41) and can be used in different predictive models for postsurgical complications (42, 43). Additionally, coagulation parameters in cancer patients that represent hemostatic and fibrinolytic systems have been proven to have association with tumor progression and dissemination (44). It has been reported that the decreased pretreatment TT was associated with the shorter esophageal squamous cell carcinoma (ESCC) and HCC survival (45, 46). Meanwhile, conventional coagulation tests after liver surgery are frequently prolonged by postoperative hypercoagulability (47), and prolonged TT before surgery indicated hyper-fibrinogenolysis, suggested as a factor that prevents the occurrence of thromboembolic complications. Other parameters that might influence perioperative complications have also been added to our models including tumor number, potassium, GGT, and TC. Although the CCIASL-pre model is applicable solely on the basis of preoperative parameters, it still appears comparable to the existing model. Despite a minor degree of discrepancy in the validation cohorts, good discriminatory performance was maintained in general. Furthermore, CCIASL-post model provides a more accurate prediction for clinicians when all postoperative indicators are available several days after operation. The CCIASL models are quite reliable because of external validation in different geographic regions.

Although the prediction models for early recurrence after liver resection of HCC had been built a lot (48–50), they are seldom derived from comprehensive candidate variables potentially relevant to HBV-related HCC. In our study, the third model for prediction of early recurrence of HCC was constructed by incorporating eight parameters from HBV-related marks, surgical conditions, pathological examination, preoperative and postoperative laboratory results. A high HBV-DNA load was identified to be an independent hepatitis-related risk for early recurrence (51, 52). In addition to HBV-related marks, the capability of laboratory findings and tumor characteristics was also evaluated in predicting early recurrence. An approach to risk stratification for early recurrence of HBV-related HCC was developed by incorporating serum AFP, tumor number, and largest tumor diameter based on the Chinese population (53). From a broader perspective of HCC studies, there is a consensus that microvascular invasion is a well-known independent prognostic factor associated with more advanced tumor stage, tumor progression, and poorer clinical outcome (54). Microvascular invasion is an essential component in the majority of current prediction models (55, 56). However, associations between these predictors were detected as well. A high HBV DNA level was an independent risk factor of microvascular invasion, and HBcAb-positive HCC was much bigger, more often involved with vascular invasion and elevated AFP (50, 56). In this case, if all possible clinical variables are not included in the candidates, it is impossible to determine the truly independent predictors. The predictive factors included in the model we built on the basis of comprehensive candidate variables are more likely to avoid this problem.

Another highlight of this article is that we discovered two significant risk factors for early metastasis, namely, short TT and absence of tumor encapsulation. Thrombin is a serine protease that performs a multifaceted role in coagulation. Previous analysis has revealed that the coagulation system has an effect on solid tumors, such as ESCC and HCC (45, 46). Zhu et al. (57) also reported that the abnormal coagulation system was an independent prognostic factor for brain metastases of non-small cell lung cancer, which is consistent with our findings in HCC. The potential mechanism may refer to the damage of liver cells, the secretion of tumor cells, and the involvement of inflammation (58, 59). Notably, capsulation of tumor was first discovered to be relevant with tumor migration.

There are still three points worth further discussion. First of all, no significant association was observed between CCI and early recurrence. Some variables included in the CCIASL models have been identified in the prediction for long-term complications but never short-term ones. In this setting, the effect of short-term complications on long-term complications still remains inconclusive. Our results provide an exploration for this topic, and no certain relationship is a preliminary conclusion. Secondly, the difference of discrimination performance between preoperative and postoperative model was insignificant according to reclassification analyses. The improvement of prediction accuracy based on postoperative data did not live up to the expectation. However, the C-index of the CCIASL-post model is much higher than that of the pre-model. Better performance is likely to be found in larger samples. Thirdly, antiviral treatment was excluded at univariable analysis of predicting high CCI, suggesting that reduction of HBV load seemed not to be helpful for prevention of severe complications. Despite a lot of evidence that long-term survival is correlated with antiviral therapy (51, 60), the association of short-term complications has never been discovered. Hence, antiviral treatment is likely to be difficult to improve clinical perioperative complications.

In summary, CCI is a well-performed measurement of postoperative complications. Our CCIASL models are clinically relevant, externally validated, and powerful tools to predict severe perioperative complications in patients with HBV-related HCC. Further prospective studies are required to explore the clinical applicability of CCIASL models in patient allocation for more frequent follow-up and clinical trials for adjuvant therapy.

## Data Availability Statement

The raw data supporting the conclusions of this article will be made available by the authors, upon reasonable request.

## Ethics Statement

This study was approved by the Ethics Committee of the First Affiliated Hospital, College of Medicine, Zhejiang University (IIT2021A0143), the First Affiliated Hospital of Xinjiang Medical University (20200116-04) and the 940th Hospital of Joint Logistics Support force of Chinese People’s Liberation Army (2021KYLL023). The patients/participants provided their written informed consent to participate in this study.

## Author Contributions

MB and QZ contributed to the conceptualization, data curation and software, formal analysis, visualization, investigation, and writing–original draft. YH contributed to funding acquisition and project administration. XyH, TA, and SW contributed to the methodology. TT, XqH, and AT contributed to the resources and validation. MB contributed to the supervision. MB and YH contributed to the writing–review and editing. All authors contributed to the article and approved the submitted version.

## Funding

This study was supported by grants from the National Natural Science Foundation of China (Grants 11971117 and 11571082) and Scientific Research Foundation of Fudan University.

## Conflict of Interest

The authors declare that the research was conducted in the absence of any commercial or financial relationships that could be construed as a potential conflict of interest.

## Publisher’s Note

All claims expressed in this article are solely those of the authors and do not necessarily represent those of their affiliated organizations, or those of the publisher, the editors and the reviewers. Any product that may be evaluated in this article, or claim that may be made by its manufacturer, is not guaranteed or endorsed by the publisher.
