# Supplementary material for: Development of Models to Predict Postoperative Complications for Hepatitis B Virus-Related Hepatocellular Carcinoma
Source: Front Oncol. 2021 Oct 5;11:717826. doi: 10.3389/fonc.2021.717826 (PMC8523990; doi:10.3389/fonc.2021.717826)
Supplement: Supplementary file 1 [file DataSheet_1.zip › Table S1 Additional information of study population.docx]

Table S1 Additional information of study population

| **Variables** | **All patients (n=675)** | **CCI≥26.2 (n=92)** | **CCI<26.2 (n=583)** | **P** |
| --- | --- | --- | --- | --- |
| **Baseline features** |  |  |  |  |
| Blood group (ABO), n (%) |  |  |  | 0.221 |
| A | 215 (31.9) | 34 (37.0) | 181 (31.1) |  |
| B | 151 (22.4) | 17 (18.5) | 134 (23.0) |  |
| O | 254 (37.7) | 30 (32.6) | 224 (38.5) |  |
| AB | 54 (8.0) | 11 (12.0) | 43 (7.4) |  |
| Blood group (RH+), n (%) | 669 (99.1) | 91 (98.9) | 578 (99.1) | 0.586 |
| Diabetes, n (%) | 64 (9.5) | 8 (8.7) | 56 (9.6) | >0.999 |
| Hypertension, n (%) | 145 (21.5) | 23 (25.0) | 122 (20.9) | 0.412 |
| Previous surgery, n (%) | 120 (17.8) | 19 (20.7) | 101 (17.3) | 0.463 |
| Alcohol assumption, n (%) | 259 (38.4) | 39 (42.4) | 220 (37.7) | 0.461 |
| Okuda stage II, n (%) | 120 (17.8) | 20 (21.7) | 100 (17.2) | 0.304 |
| ASA score, n (%) |  |  |  | 0.009 |
| I | 181 (26.8) | 19 (20.7) | 162 (27.8) |  |
| II | 450 (66.7) | 60 (65.2) | 390 (66.9) |  |
| III | 44 (6.5) | 13 (14.1) | 31 (5.3) |  |
| **Radiology findings** |  |  |  |  |
| Peripheral tumor, n (%) | 361 (53.6) | 45 (48.9) | 316 (54.4) | 0.368 |
| Portal vein phase decrease, n (%) | 639 (94.9) | 89 (96.7) | 550 (94.7) | 0.607 |
| Lag phase decrease, n (%) | 633 (94.2) | 86 (93.5) | 547 (94.3) | 0.809 |
| Washout, n (%) | 620 (92.3) | 85 (92.4) | 535 (92.2) | >0.999 |
| Lymphadenectasis, n (%) | 178 (26.4) | 25 (27.2) | 153 (26.3) | 0.899 |
| Esophageal varices, n (%) | 39 (5.8) | 5 (5.4) | 34 (5.9) | >0.999 |
| **Surgical features** |  |  |  |  |
| Da Vinci robotic-assisted surgery, n (%) | 4 (0.6) | 0 (0.0) | 4 (0.7) | >0.999 |
| Cholecystectomy, n (%) | 309 (46.0) | 43 (46.7) | 266 (45.9) | 0.911 |
| Splenomegaly, n (%) | 282 (41.9) | 37 (40.2) | 245 (42.2) | 0.820 |
| **Pathologic Characteristics** |  |  |  |  |
| Tumor giant cell, n (%) | 47 (7.0) | 6 (6.5) | 41 (7.1) | >0.999 |
| Surrounding liver tissue, n (%) |  |  |  | 0.940 |
| Normal | 211 (31.4) | 32 (34.8) | 179 (30.8) |  |
| Cirrhosis | 365 (54.2) | 47 (51.1) | 318 (54.7) |  |
| Fibrosis | 34 (5.1) | 5 (5.4) | 29 (5.0) |  |
| Fatty degeneration | 15 (2.2) | 2 (2.2) | 13 (2.2) |  |
| Inflammation | 48 (7.1) | 6 (6.5) | 42 (7.2) |  |
| Tumor encapsulation (%) |  |  |  | 0.232 |
| Complete | 397 (59.0) | 56 (60.9) | 341 (58.7) |  |
| Incomplete | 197 (29.3) | 30 (32.6) | 167 (28.7) |  |
| Absent | 79 (11.7) | 6 (6.5) | 73 (12.6) |  |
| Cytokeratin 7, n (%) | 211 (41.5) | 27 (41.5) | 184 (41.5) | >0.999 |
| Glypican-3, n (%) | 537 (89.2) | 71 (91.0) | 466 (88.9) | 0.698 |
| **Preoperative laboratory findings, median [IQR]** |  |  |  |  |
| Neutrophil, % | 59.90 [53.10, 66.45] | 61.05 [55.60, 65.60] | 59.70 [52.55, 66.50] | 0.307 |
| Lymphocyte, % | 28.95 [23.30, 35.58] | 27.70 [22.87, 33.85] | 29.20 [23.60, 36.03] | 0.065 |
| Monocyte, % | 7.30 [5.90, 9.10] | 8.00 [6.88, 10.20] | 7.20 [5.73, 9.00] | 0.001 |
| Eosinophil, % | 2.10 [1.20, 3.40] | 2.30 [1.08, 3.42] | 2.00 [1.20, 3.40] | 0.709 |
| Hemoglobin, g/L | 145.00 [132.00, 155.00] | 139.00 [126.00, 153.00] | 145.50 [132.00, 155.75] | 0.010 |
| Platelets, 10^9/L | 147.50 [113.00, 196.75] | 141.50 [99.25, 198.25] | 148.50 [114.00, 195.75] | 0.311 |
| Urine specific gravity | 1.02 [1.01, 1.02] | 1.02 [1.01, 1.02] | 1.02 [1.01, 1.02] | 0.399 |
| Total Protein, g/L | 70.00 [65.50, 73.80] | 68.25 [64.97, 71.73] | 70.40 [65.80, 74.00] | 0.019 |
| Albumin, g/L | 42.15 [39.10, 45.50] | 41.35 [38.70, 44.10] | 42.30 [39.10, 45.80] | 0.032 |
| Globulin, g/L | 27.40 [24.80, 30.50] | 27.35 [24.87, 30.72] | 27.40 [24.80, 30.40] | 0.741 |
| Alkaline phosphatase, U/L | 88.00 [72.00, 112.00] | 97.50 [75.75, 126.50] | 87.00 [71.00, 110.00] | 0.012 |
| Cholinesterase, U/L | 6686.50 [5492.00, 7935.75] | 6240.00 [5144.75, 7413.00] | 6775.00 [5585.50, 7988.50] | 0.010 |
| Total bile acid, μmol/L | 8.00 [5.00, 13.00] | 8.00 [5.00, 12.25] | 8.00 [5.00, 13.00] | 0.848 |
| Direct bilirubin, μmol/L | 5.00 [4.00, 6.00] | 5.00 [4.00, 7.00] | 5.00 [4.00, 6.00] | 0.017 |
| Indirect bilirubin, μmol/L | 8.00 [6.00, 11.00] | 8.90 [6.00, 11.00] | 8.00 [6.00, 11.00] | 0.595 |
| Adenosine deaminase, U/L | 13.00 [10.00, 17.00] | 15.00 [11.00, 18.00] | 13.00 [10.00, 17.00] | 0.060 |
| Glomerular filtration rate, mL/min | 96.30 [86.75, 105.48] | 96.62 [86.60, 104.71] | 96.26 [86.99, 105.60] | 0.958 |
| Creatinine, umol/L | 73.00 [64.00, 82.00] | 73.00 [66.75, 81.00] | 73.00 [64.00, 83.00] | 0.873 |
| Triglyceride, mmol/L | 0.93 [0.74, 1.23] | 0.88 [0.74, 1.14] | 0.94 [0.74, 1.24] | 0.257 |
| High-density lipoprotein, mmol/L | 1.12 [0.93, 1.35] | 1.09 [0.90, 1.30] | 1.13 [0.94, 1.38] | 0.153 |
| Low-density lipoprotein, mmol/L | 2.25 [1.82, 2.75] | 2.13 [1.58, 2.74] | 2.26 [1.88, 2.75] | 0.036 |
| Very low-density lipoprotein, mmol/L | 0.49 [0.35, 0.68] | 0.49 [0.33, 0.69] | 0.50 [0.35, 0.68] | 0.778 |
| Fasting blood glucose, mmol/L | 4.87 [4.42, 5.46] | 4.76 [4.45, 5.63] | 4.87 [4.42, 5.44] | 0.581 |
| Sodium, mmol/L | 141.00 [140.00, 143.00] | 141.00 [140.00, 143.00] | 141.00 [140.00, 143.00] | 0.707 |
| α-L-fucosidase, U/L | 32.00 [26.00, 41.00] | 32.00 [27.00, 40.00] | 32.00 [26.00, 41.00] | 0.881 |
| International normalized ratio | 1.03 [0.98, 1.09] | 1.05 [1.00, 1.10] | 1.03 [0.98, 1.09] | 0.053 |
| Fibrinogen, g/L | 2.54 [2.06, 3.14] | 2.71 [2.14, 3.53] | 2.50 [2.05, 3.10] | 0.070 |
| Activated partial thromboplastin time, s | 28.60 [26.10, 31.70] | 29.00 [25.95, 32.28] | 28.60 [26.10, 31.58] | 0.712 |
| Prothrombin time, s | 11.80 [11.30, 12.50] | 12.05 [11.50, 12.60] | 11.80 [11.30, 12.40] | 0.052 |
| D-dimer, μg/L | 347.50 [182.75, 735.50] | 408.50 [181.50, 952.75] | 343.00 [183.50, 690.00] | 0.200 |
| α-fetoprotein, ng/mL * | 5.77 [2.38, 10.36] | 5.54 [2.58, 10.34] | 5.77 [2.32, 10.36] | 0.610 |
| Carcinoembryonic antigen, ng/mL | 2.40 [1.60, 3.70] | 2.50 [1.60, 3.80] | 2.40 [1.70, 3.70] | 0.666 |
| Carbohydrate antigen199, U/mL * | 3.02 [2.10, 3.92] | 3.63 [2.32, 4.20] | 2.96 [2.07, 3.89] | 0.023 |
| Carbohydrate antigen125, U/mL * | 3.63 [3.05, 4.35] | 3.78 [3.10, 4.61] | 3.62 [3.05, 4.29] | 0.251 |
| Ferritin, ng/mL * | 7.79 [7.01, 8.55] | 7.99 [7.17, 8.93] | 7.75 [6.99, 8.49] | 0.039 |
| Total prostate-specific antigen, ng/mL | 0.67 [0.42, 1.24] | 0.69 [0.44, 1.17] | 0.66 [0.42, 1.24] | 0.948 |
| Hepatitis B core antibody-IgM, S/CO | 0.10 [0.10, 0.19] | 0.10 [0.10, 0.18] | 0.10 [0.10, 0.19] | 0.931 |
| Urine protein, n (%) |  |  |  | 0.027 |
| 0 | 450 (67.1) | 54 (58.7) | 396 (68.4) |  |
| 1 | 214 (31.9) | 35 (38.0) | 179 (30.9) |  |
| 2 | 4 (0.6) | 1 (1.1) | 3 (0.5) |  |
| 3 | 2 (0.3) | 1 (1.1) | 1 (0.2) |  |
| 4 | 1 (0.1) | 1 (1.1) | 0 (0.0) |  |
| Urine bilirubin, n (%) |  |  |  | 0.294 |
| 0 | 666 (99.3) | 91 (98.9) | 575 (99.3) |  |
| 1 | 3 (0.4) | 0 (0.0) | 3 (0.5) |  |
| 2 | 1 (0.1) | 1 (1.1) | 0 (0.0) |  |
| 3 | 1 (0.1) | 0 (0.0) | 1 (0.2) |  |
| Urine ketone, n (%) |  |  |  | 0.782 |
| 0 | 650 (96.9) | 89 (96.7) | 561 (96.9) |  |
| 1 | 20 (3.0) | 3 (3.3) | 17 (2.9) |  |
| 2 | 1 (0.1) | 0 (0.0) | 1 (0.2) |  |
| Urobilinogen, n (%) |  |  |  | 0.261 |
| 0 | 552 (82.4) | 71 (77.2) | 481 (83.2) |  |
| 1 | 86 (12.8) | 14 (15.2) | 72 (12.5) |  |
| 2 | 21 (3.1) | 4 (4.3) | 17 (2.9) |  |
| 3 | 9 (1.3) | 3 (3.3) | 6 (1.0) |  |
| 4 | 2 (0.3) | 0 (0.0) | 2 (0.3) |  |
| **Postoperative laboratory findings, median [IQR]** |  |  |  |  |
| Neutrophil, % | 71.90 [66.40, 77.00] | 74.40 [68.20, 78.60] | 71.70 [66.10, 76.80] | 0.014 |
| Lymphocyte, % | 15.10 [11.30, 18.90] | 13.60 [9.67, 17.50] | 15.30 [11.50, 19.00] | 0.008 |
| Monocyte, % | 9.40 [7.60, 11.90] | 8.65 [6.90, 11.38] | 9.50 [7.70, 12.10] | 0.060 |
| Eosinophil, % | 2.30 [1.30, 3.60] | 2.20 [1.28, 3.60] | 2.30 [1.30, 3.60] | 0.986 |
| Hemoglobin, g/L | 112.00 [99.00, 124.00] | 102.50 [92.75, 118.00] | 113.00 [100.00, 125.00] | 0.002 |
| Platelets, 10^9/L | 138.00 [99.00, 194.00] | 132.00 [101.75, 193.25] | 139.00 [99.00, 194.00] | 0.675 |
| Total Protein, g/L | 58.00 [54.30, 62.23] | 56.20 [52.15, 60.92] | 58.15 [54.60, 62.40] | 0.007 |
| Albumin, g/L | 35.00 [32.50, 37.82] | 33.85 [30.82, 37.42] | 35.15 [32.50, 37.92] | 0.030 |
| Globulin, g/L | 23.00 [20.28, 25.72] | 22.00 [18.78, 25.15] | 23.10 [20.30, 25.92] | 0.057 |
| Albumin-globulin ratio | 1.50 [1.37, 1.70] | 1.60 [1.37, 1.80] | 1.50 [1.37, 1.70] | 0.678 |
| Alkaline phosphatase, U/L | 80.00 [65.00, 102.00] | 86.00 [69.00, 124.00] | 79.00 [65.00, 100.00] | 0.012 |
| Cholinesterase, U/L | 3354.00 [2710.00, 4186.25] | 3123.00 [2338.00, 3646.00] | 3422.00 [2748.00, 4293.00] | <0.001 |
| Total bile acid, μmol/L | 7.25 [5.00, 13.00] | 7.00 [5.00, 20.00] | 7.60 [5.00, 13.00] | 0.236 |
| Direct bilirubin, μmol/L | 9.00 [6.00, 13.00] | 11.00 [8.00, 19.25] | 9.00 [6.00, 13.00] | <0.001 |
| Indirect bilirubin, μmol/L | 10.00 [7.00, 14.00] | 11.50 [8.00, 17.25] | 10.00 [7.00, 13.00] | 0.002 |
| Adenosine deaminase, U/L | 10.00 [7.00, 12.00] | 10.00 [8.00, 13.00] | 9.60 [7.00, 12.00] | 0.111 |
| Glomerular filtration rate, mL/min | 106.09 [95.34, 117.08] | 103.64 [93.39, 115.43] | 106.29 [96.11, 117.14] | 0.119 |
| Creatinine, μmol/L | 62.00 [54.00, 73.00] | 63.00 [54.50, 75.00] | 62.00 [53.00, 72.00] | 0.174 |
| Triglyceride, mmol/L | 0.86 [0.68, 1.09] | 0.88 [0.74, 1.08] | 0.86 [0.68, 1.09] | 0.206 |
| High-density lipoprotein, mmol/L | 0.59 [0.46, 0.76] | 0.49 [0.35, 0.64] | 0.61 [0.47, 0.77] | <0.001 |
| Low-density lipoprotein, mmol/L | 1.38 [1.03, 1.76] | 1.20 [0.86, 1.77] | 1.39 [1.07, 1.75] | 0.052 |
| Very low-density lipoprotein, mmol/L | 0.56 [0.43, 0.72] | 0.62 [0.45, 0.76] | 0.55 [0.43, 0.72] | 0.074 |
| Fasting blood glucose, mmol/L | 4.92 [4.40, 5.67] | 4.92 [4.35, 5.97] | 4.92 [4.41, 5.64] | 0.897 |
| **Perioperative situation (median [IQR])** |  |  |  |  |
| POD1 drainage of gastrointestinal decompression, mL, median [IQR] | 30.00 [10.00, 80.00] | 30.00 [10.00, 55.00] | 30.00 [10.00, 80.00] | 0.661 |
| POD 3 ascites volume, mL, median [IQR] | 70.00 [20.00, 150.00] | 80.00 [15.00, 200.00] | 70.00 [20.00, 120.00] | 0.309 |

POD: postoperative day; IQR: interquartile range. * data was natural log transformed.
