# Supplementary material for: Development of Models to Predict Postoperative Complications for Hepatitis B Virus-Related Hepatocellular Carcinoma
Source: Front Oncol. 2021 Oct 5;11:717826. doi: 10.3389/fonc.2021.717826 (PMC8523990; doi:10.3389/fonc.2021.717826)
Supplement: Supplementary file 1 [file DataSheet_1.zip › Table S2 Early recurrence predictors selection.docx]

Table S2 Uni- and multivariate Cox regression analysis of predictors for early recurrence

|  | **Univariate analysis** | | | **Multivariate analysis** | | |
| --- | --- | --- | --- | --- | --- | --- |
| **Variables** | **HR** | **95% CI** | **P** | **HR** | **95% CI** | **P** |
| **Baseline factors** | | | | | | |
| *CCI (high vs low)* | *0.862* | *0.563-1.321* | *0.496* |  |  |  |
| Sex (male vs female) | 1.091 | 0.751-1.585 | 0.649 |  |  |  |
| Age (years) | 1.005 | 1.002-1.007 | 0.001 |  |  |  |
| NNIS index |  |  |  |  |  |  |
| 1 vs 0 | 1.109 | 0.811-1.517 | 0.516 |  |  |  |
| 2 vs 0 | 2.291 | 1.043-5.032 | 0.039 |  |  |  |
| ASA score |  |  |  |  |  |  |
| II vs I | 1.059 | 0.773-1.450 | 0.720 |  |  |  |
| III vs I | 1.116 | 0.640-1.945 | 0.699 |  |  |  |
| BCLC stage |  |  |  |  |  |  |
| A1 vs 0 | 1.093 | 0.779-1.532 | 0.607 |  |  |  |
| A2 vs 0 | 1.002 | 0.248-4.052 | 0.997 |  |  |  |
| A3 vs 0 | 0.996 | 0.608-1.632 | 0.987 |  |  |  |
| A4 vs 0 | 1.428 | 0.699-2.919 | 0.328 |  |  |  |
| **Hepatitis B marks** | | | | | | |
| HBV DNA (IU/ml) | 1.031 | 1.014-1.048 | <0.001 | 1.024 | 1.006-1.042 | 0.009 |
| HBsAg (IU/ml) | 1.002 | 1.001-1.003 | 0.002 |  |  |  |
| HBeAg (S/CO) | 1.002 | 1.001-1.003 | 0.001 |  |  |  |
| HBcAb (S/CO) | 0.989 | 0.949-1.030 | 0.586 |  |  |  |
| HBeAb (S/CO) | 1.044 | 1.018-1.071 | 0.001 |  |  |  |
| HBsAb (IU/ml) | 1.003 | 0.998-1.009 | 0.257 |  |  |  |
| **Tumor characteristics** | | | | | | |
| Arterial phase  (steady vs enhancement) | 0.323 | 0.133-0.787 | 0.013 | 0.302 | 0.121-0.755 | 0.010 |
| Tumor encapsulation |  |  |  |  |  |  |
| Incomplete vs Complete | 1.495 | 1.100-2.031 | 0.010 |  |  |  |
| Absent vs Complete | 1.735 | 1.123-2.681 | 0.013 |  |  |  |
| Surrounding satellite nodules (present vs absent) | 2.022 | 1.454-2.814 | <0.001 | 2.010 | 1.419-2.847 | <0.001 |
| Maximum tumor size (cm) | 1.086 | 1.041-1.132 | <0.001 |  |  |  |
| Microvascular invasion (present vs absent) | 2.005 | 1.518-2.650 | <0.001 | 1.699 | 1.268-2.275 | <0.001 |
| Hepatic capsule |  |  |  |  |  |  |
| Invaded vs Normal | 1.593 | 1.058-2.397 | 0.026 |  |  |  |
| Attached vs Normal | 1.113 | 0.665-1.862 | 0.683 |  |  |  |
| **Preoperative laboratory results** | | | | | | |
| Platelets (10^9/L) | 0.999 | 0.997-1.001 | 0.499 |  |  |  |
| Total Protein (g/L) | 0.975 | 0.954-0.996 | 0.023 | 0.979 | 0.959-1.000 | 0.048 |
| Alanine aminotransferase (U/L) | 1.004 | 1.001-1.006 | 0.001 |  |  |  |
| Aspartate aminotransferase (U/L) | 1.003 | 1.001-1.005 | 0.003 |  |  |  |
| Alkaline phosphatase (U/L) | 1.002 | 0.999-1.005 | 0.205 |  |  |  |
| Total bilirubin (μmol/L) | 1.040 | 1.012-1.068 | 0.004 |  |  |  |
| Direct bilirubin (μmol/L) | 1.159 | 1.093-1.228 | <0.001 | 1.148 | 1.081-1.220 | <0.001 |
| α-L-fucosidase (U/L) | 1.014 | 1.005-1.023 | 0.003 |  |  |  |
| α-fetoprotein (μg/L) | 1.050 | 1.020-1.081 | 0.001 |  |  |  |
| **Postoperative laboratory results** | | | | | | |
| Platelets (10^9/L) | 1.002 | 1.000-1.005 | 0.032 | 1.002 | 1.000-1.005 | 0.046 |
| Total Protein (g/L) | 1.006 | 0.983-1.030 | 0.621 |  |  |  |
| Alanine aminotransferase (U/L) | 1.000 | 0.998-1.002 | 0.805 |  |  |  |
| Aspartate aminotransferase (U/L) | 1.000 | 0.998-1.003 | 0.721 |  |  |  |
| Alkaline phosphatase (U/L) | 1.004 | 1.001-1.007 | 0.003 | 1.004 | 1.000-1.007 | 0.034 |
| Total bilirubin (μmol/L) | 1.007 | 0.998-1.015 | 0.117 |  |  |  |
| Direct bilirubin (μmol/L) | 1.013 | 1.003-1.023 | 0.013 |  |  |  |
