# Supplementary material for: Development of Models to Predict Postoperative Complications for Hepatitis B Virus-Related Hepatocellular Carcinoma
Source: Front Oncol. 2021 Oct 5;11:717826. doi: 10.3389/fonc.2021.717826 (PMC8523990; doi:10.3389/fonc.2021.717826)
Supplement: Supplementary file 1 [file DataSheet_1.zip › Table S3 Subgroup analysis.docx]

Table S3 Subgroup analysis of comprehensive complication index (CCI) on early recurrence

| **Characteristics** | **CCI< 26.2** | **CCI≥26.2** | **HR (95%CI)** | **P** |
| --- | --- | --- | --- | --- |
| **All patients** | 360 | 55 | 0.862 (0.563-1.321) | 0.496 |
| **Antiviral treatment** | |  |  |  |
| No | 265 (73.61) | 41 (74.55) | 0.925 (0.572-1.497) | 0.752 |
| Yes | 95 (26.39) | 14 (25.45) | 0.691 (0.273-1.746) | 0.434 |
| **Platelets (10^9/L)** | |  |  |  |
| >100 | 294 (81.67) | 41 (74.55) | 0.991 (0.62-1.582) | 0.968 |
| <100 | 66 (18.33) | 14 (25.45) | 0.517 (0.183-1.459) | 0.213 |
| **ALT (U/L)** |  |  |  |  |
| <44 | 272 (75.56) | 39 (70.91) | 0.909 (0.548-1.509) | 0.712 |
| >44 | 88 (24.44) | 16 (29.09) | 0.735 (0.333-1.621) | 0.445 |
| **AST (U/L)** |  |  |  |  |
| <40 | 243 (67.50) | 37 (67.27) | 1.114 (0.667-1.862) | 0.679 |
| >40 | 117 (32.50) | 18 (32.73) | 0.531 (0.244-1.153) | 0.110 |
| **HBV DNA (copies)** | |  |  |  |
| <2000 | 151 (41.94) | 22 (40.00) | 0.599 (0.259-1.386) | 0.231 |
| >2000 | 209 (58.06) | 33 (60.00) | 1.004 (0.611-1.65) | 0.988 |
| **AFP (ug/L)** |  |  |  |  |
| <40 | 172 (48.45) | 31 (56.36) | 1.171 (0.646-2.122) | 0.604 |
| >40 | 183 (51.55) | 24 (43.64) | 0.696 (0.374-1.295) | 0.252 |
| **HBeAg** |  |  |  |  |
| Negative | 262 (72.78) | 40 (72.73) | 1.014 (0.617-1.667) | 0.955 |
| Positive | 98 (27.22) | 15 (27.27) | 0.570 (0.246-1.319) | 0.189 |
| **HBeAb** |  |  |  |  |
| Negative | 222 (61.67) | 37 (67.27) | 1.114 (0.674-1.84) | 0.673 |
| Positive | 138 (38.33) | 18 (32.73) | 0.517 (0.225-1.187) | 0.120 |
| **Surgical approach** | |  |  |  |
| Minimally invasive | 58 (16.25) | 3 (5.45) | 0.858 (0.116-6.372) | 0.881 |
| Open | 299 (83.75) | 52 (94.55) | 0.827 (0.533-1.282) | 0.395 |
| **Intraoperative blood loss (mL)** | | |  |  |
| <1000 | 349 (96.94) | 51 (92.73) | 0.887 (0.568-1.383) | 0.595 |
| >1000 | 11 (3.06) | 4 (7.27) | 0.366 (0.076-1.763) | 0.210 |
| **Intraoperative blood transfusion** | | |  |  |
| No | 337 (94.13) | 48 (88.89) | 0.890 (0.565-1.403) | 0.616 |
| Yes | 21 (5.87) | 6 (11.11) | 0.371 (0.083-1.651) | 0.193 |
| **Tumor number** | |  |  |  |
| Solitary | 321 (89.42) | 41 (74.55) | 0.853 (0.524-1.39) | 0.524 |
| Multiple | 38 (10.58) | 14 (25.45) | 0.842 (0.338-2.097) | 0.712 |
| **Satellites** |  |  |  |  |
| Absent | 310 (86.11) | 47 (85.45) | 0.781 (0.478-1.275) | 0.323 |
| Present | 50 (13.89) | 8 (14.55) | 1.326 (0.554-3.172) | 0.527 |
| **Pringle Maneuver** | |  |  |  |
| No | 311 (86.39) | 49 (89.09) | 0.790 (0.495-1.26) | 0.323 |
| Yes | 49 (13.61) | 6 (10.91) | 1.726 (0.602-4.952) | 0.310 |
| **MVI** |  |  |  |  |
| Absent | 211 (58.61) | 33 (60.00) | 0.823 (0.439-1.542) | 0.543 |
| Present | 149 (41.39) | 22 (40.00) | 0.888 (0.496-1.589) | 0.689 |
| **Maximum tumor size (cm)** | |  |  |  |
| <5 | 187 (51.94) | 24 (43.64) | 0.852 (0.428-1.695) | 0.647 |
| >5 | 173 (48.06) | 31 (56.36) | 0.818 (0.474-1.413) | 0.472 |
| **Tumor differentiation** | |  |  |  |
| High | 4 (1.12) | 0 (0.00) |  |  |
| Moderate | 18 (5.04) | 5 (9.09) | 1.864 (0.169-20.601) | 0.612 |
| Moderate to Poor | 295 (82.63) | 46 (83.64) | 0.814 (0.51-1.298) | 0.387 |
| Poor | 40 (11.20) | 4 (7.27) | 1.42 (0.423-4.766) | 0.570 |
| **BCLC stage** |  |  |  |  |
| 0 | 234 (65.00) | 31 (56.36) | 1.128 (0.667-1.906) | 0.653 |
| A1 | 84 (23.33) | 10 (18.18) | 0.29 (0.07-1.198) | 0.087 |
| A2 | 4 (1.11) | 0 (0.00) |  |  |
| A3 | 29 (8.06) | 9 (16.36) | 1.110 (0.365-3.379) | 0.854 |
| A4 | 9 (2.50) | 5 (9.09) | 0.457 (0.092-2.278) | 0.340 |

ALT: Alanine aminotransferase; AST: Aspartate aminotransferase; AFP: α -fetoprotein; HBV: Hepatitis B Virus; HBeAg: Hepatitis B e antigen; HBeAB: Hepatitis B e antibody; MVI: Microvascular invasion; BCLC: Barcelona Clinic Liver Cancer; HR: Hazard ratio; CI: Confidence interval.
