# Supplementary material for: Development of Models to Predict Postoperative Complications for Hepatitis B Virus-Related Hepatocellular Carcinoma
Source: Front Oncol. 2021 Oct 5;11:717826. doi: 10.3389/fonc.2021.717826 (PMC8523990; doi:10.3389/fonc.2021.717826)
Supplement: Supplementary file 1 [file DataSheet_1.zip › Table S4 Distribution of metastasis location.docx]

Table S4 Distribution of metastatic location in entire population

|  | Internal dataset (n=415) | | External dataset (n=372) | |
| --- | --- | --- | --- | --- |
| Location | Cases | Proportion of entire population (%) | Cases | Proportion of entire population (%) |
| Lung | 30 | 7.23 | 36 | 9.68 |
| lymphonodus | 4 | 0.96 | 37 | 9.95 |
| Liver | 7 | 1.67 | 119 | 32.0 |
| Abdomen | 9 | 2.22 | 18 | 4.84 |
| Bone | 9 | 2.17 | 12 | 3.23 |
| Brain | 3 | 0.72 | 1 | 0.27 |
| Total | 62 | 14.9 | 223 | 59.9 |
