# Supplementary material for: Development of Models to Predict Postoperative Complications for Hepatitis B Virus-Related Hepatocellular Carcinoma
Source: Front Oncol. 2021 Oct 5;11:717826. doi: 10.3389/fonc.2021.717826 (PMC8523990; doi:10.3389/fonc.2021.717826)
Supplement: Supplementary file 1 [file DataSheet_1.zip › Table S5 Univariate GLM selection and GEE.docx]

Table S5 Univariate GLM selection and GEE internal validation of variables associated with metastasis

|  | **Univariate GLM Selection** | | | **GEE Internal Validation** | | |
| --- | --- | --- | --- | --- | --- | --- |
| **Variables** | **OR** | **95% CI** | **P** | **OR** | **95% CI** | **P** |
| *CCI (high vs low)* | *0.759* | *0.327-1.761* | *0.521* |  |  |  |
| Basophil (%) | 0.280 | 0.087-0.903 | 0.033 | 0.649 | 0.167-2.538 | 0.533 |
| HDL (mmol/L) | 0.414 | 0.177-0.972 | 0.043 | 1.304 | 0.620-2.745 | 0.484 |
| LDL (mmol/L) | 1.433 | 1.052-1.953 | 0.023 | 1.121 | 0.825-1.524 | 0.465 |
| Fasting blood glucose (mmol/L) | 0.759 | 0.579-0.996 | 0.046 | 0.696 | 0.518-0.934 | 0.016 |
| α-L-fucosidase (u/g) | 1.020 | 1.002-1.037 | 0.027 | 1.001 | 0.982-1.020 | 0.951 |
| Fibrinogen (g/L) | 1.512 | 1.176-1.943 | 0.001 | 1.083 | 0.808-1.452 | 0.594 |
| Thrombin time (s) | 0.776 | 0.640-0.941 | 0.010 | 0.771 | 0.623-0.953 | 0.016 |
| CA199 (U/mL) | 1.290 | 1.010-1.649 | 0.041 | 0.963 | 0.745-1.246 | 0.777 |
| Ferritin (ug/L) | 1.562 | 1.144-2.133 | 0.005 | 1.297 | 0.921-1.826 | 0.137 |
| Intraoperative blood loss (mL) | 1.002 | 1.001-1.002 | <0.001 | 1.001 | 1.000-1.002 | 0.053 |
| Postoperative platelet (10^9/L) | 1.004 | 1.000-1.008 | 0.041 | 1.000 | 0.995-1.005 | 0.912 |
| Postoperative total protein (g/L) | 1.064 | 1.019-1.112 | 0.005 | 1.079 | 1.002-1.163 | 0.045 |
| Postoperative albumin (g/L) | 1.089 | 1.017-1.166 | 0.014 | 0.986 | 0.880-1.104 | 0.803 |
| Maximum tumor size (cm) | 1.190 | 1.103-1.283 | <0.001 | 1.082 | 0.999-1.172 | 0.053 |
| Surrounding satellite nodules (present vs absent) | 2.667 | 1.455-4.888 | 0.002 | 1.962 | 1.060-3.630 | 0.032 |
| MVI (present vs absent) | 1.836 | 1.077-3.128 | 0.025 | 1.039 | 0.578-1.868 | 0.897 |
| NNIS index |  |  |  |  |  |  |
| 1 vs 0 | 2.114 | 1.061-4.213 | 0.033 | 1.337 | 0.698-2.559 | 0.381 |
| 2 vs 0 | 2.753 | 0.508-14.92 | 0.240 | 1.062 | 0.170-6.623 | 0.949 |
| Tumor encapsulation |  |  |  |  |  |  |
| Incomplete vs Complete | 1.739 | 0.964-3.137 | 0.066 | 1.376 | 0.747-2.553 | 0.306 |
| Absent vs Complete | 2.753 | 1.251-6.061 | 0.012 | 4.111 | 1.921-8.795 | <0.001 |
| Hepatic capsule |  |  |  |  |  |  |
| Invaded vs Normal | 2.529 | 1.211-5.282 | 0.014 | 0.987 | 0.452-2.154 | 0.974 |
| Attached vs Normal | 2.091 | 0.888-4.920 | 0.091 | 1.559 | 0.721-3.547 | 0.248 |

CCI: Comprehensive complication index; HDL: high-density lipoprotein; LDL: low-density lipoprotein; CA199: Carbohydrate antigen 199; MVI: Microvascular invasion; NNIS: National Nosocomial Infection Surveillance; GLM: Generalized linear model; GEE: Generalized estimation equation; OR: Odds ratio; CI: Confidence interval.
