# Supplementary material for: Development of Models to Predict Postoperative Complications for Hepatitis B Virus-Related Hepatocellular Carcinoma
Source: Front Oncol. 2021 Oct 5;11:717826. doi: 10.3389/fonc.2021.717826 (PMC8523990; doi:10.3389/fonc.2021.717826)
Supplement: Supplementary file 1 [file DataSheet_1.zip › Table S6 GEE working correlation matrix.docx]

Table S6 GEE working correlation matrix

| **Internal matrix** |  |  |  |  |  |  |
| --- | --- | --- | --- | --- | --- | --- |
| Location | Lung | lymphonodus | Liver | Abdomen | Bone | Brain |
| Lung | 1 |  |  |  |  |  |
| lymphonodus | -0.062 | 1 |  |  |  |  |
| Liver | -0.041 | 0 | 1 |  |  |  |
| Abdomen | 0.065 | -0.008 | -0.017 | 1 |  |  |
| Bone | -0.064 | 0.009 | -0.018 | -0.01 | 1 |  |
| Brain | -0.076 | 0.013 | 0.004 | -0.004 | -0.005 | 1 |
| **External matrix** |  |  |  |  |  |  |
| Location | Lung | lymphonodus | Liver | Abdomen | Bone | Brain |
| Lung | 1 |  |  |  |  |  |
| lymphonodus | -0.062 | 1 |  |  |  |  |
| Liver | 0.198 | 0.098 | 1 |  |  |  |
| Abdomen | 0.187 | 0.087 | -0.046 | 1 |  |  |
| Bone | 0.052 | 0.01 | -0.092 | 0.074 | 1 |  |
| Brain | 0.042 | 0.008 | -0.236 | 0.072 | 0.093 | 1 |
