# Supplementary figures and images for: Development of Models to Predict Postoperative Complications for Hepatitis B Virus-Related Hepatocellular Carcinoma
Source: Front Oncol. 2021 Oct 5;11:717826. doi: 10.3389/fonc.2021.717826 (PMC8523990; doi:10.3389/fonc.2021.717826)

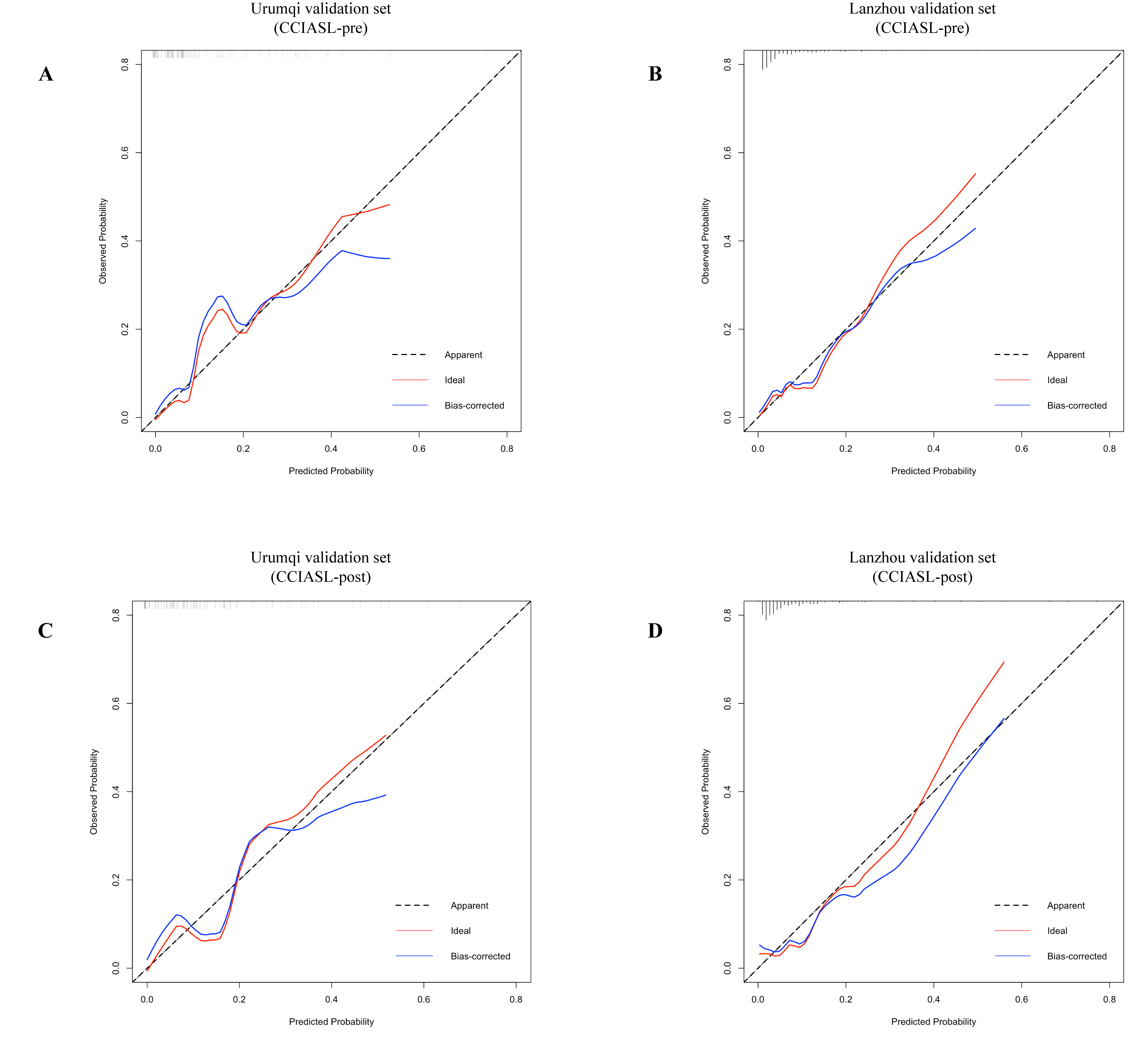

Supplement: Supplementary file 1 [file DataSheet_1.zip › Figure S1.tif]

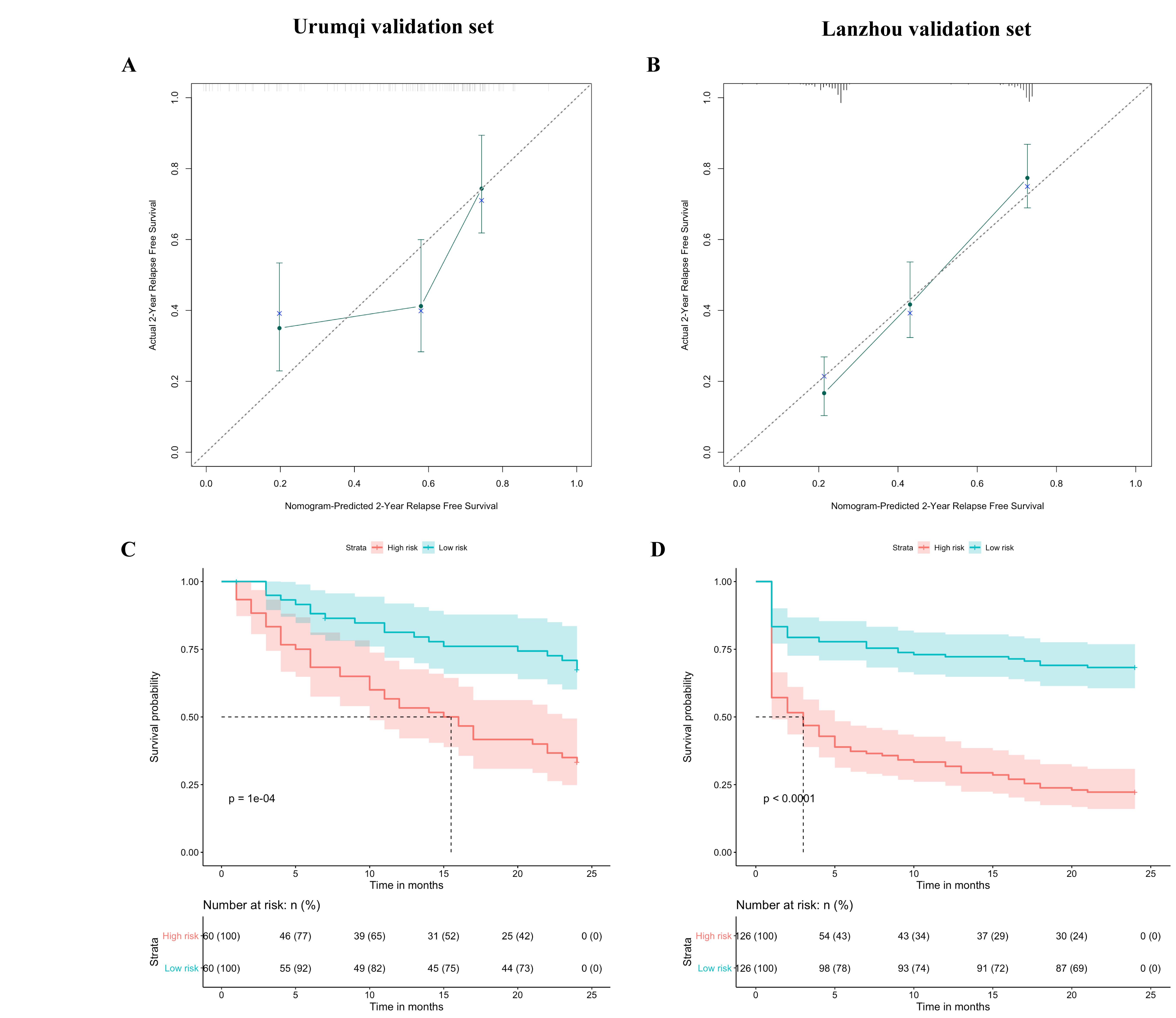

Supplement: Supplementary file 1 [file DataSheet_1.zip › Figure S2.tif]
